# A Comparative Characterization of Different Host-sourced *Lactobacillus ruminis* Strains and Their Adhesive, Inhibitory, and Immunomodulating Functions

**DOI:** 10.3389/fmicb.2017.00657

**Published:** 2017-04-13

**Authors:** Xia Yu, Silja Åvall-Jääskeläinen, Joanna Koort, Agneta Lindholm, Johanna Rintahaka, Ingemar von Ossowski, Airi Palva, Ulla Hynönen

**Affiliations:** Department of Veterinary Biosciences, Faculty of Veterinary Medicine, University of HelsinkiHelsinki, Finland

**Keywords:** *Lactobacillus ruminis*, adhesion, inhibition of pathogens, barrier function, TLRs

## Abstract

*Lactobacillus ruminis*, an autochthonous member of the gastrointestinal microbiota of humans and many animals, is a less characterized but interesting species for many reasons, including its intestinal prevalence and possible positive roles in host–microbe crosstalk. In this study, we isolated a novel *L. ruminis* strain (GRL 1172) from porcine feces and analyzed its functional characteristics and niche adaptation factors in parallel with those of three other *L. ruminis* strains (a human isolate, ATCC 25644, and two bovine isolates, ATCC 27780 and ATCC 27781). All the strains adhered to fibronectin, type I collagen, and human colorectal adenocarcinoma cells (HT-29), but poorly to type IV collagen, porcine intestinal epithelial cells (IPEC-1), and human colon adenocarcinoma cells (Caco-2). In competition assays, all the strains were able to inhibit the adhesion of *Yersinia enterocolitica* and enterotoxigenic *Escherichia coli* (ETEC, F4^+^) to fibronectin, type I; collagen, IPEC-1, and Caco-2 cells, and the inhibition rates tended to be higher than in exclusion assays. The culture supernatants of the tested strains inhibited the growth of six selected pathogens to varying extents. The inhibition was solely based on the low pH resulting from acid production during growth. All four *L. ruminis* strains supported the barrier function maintenance of Caco-2 cells, as shown by the modest increase in *trans*-epithelial electrical resistance and the prevention of dextran diffusion during co-incubation. However, the strains could not prevent the barrier damage caused by ETEC in the Caco-2 cell model. All the tested strains and their culture supernatants were able to provoke Toll-like receptor (TLR) 2-mediated NF-κB activation and IL-8 production *in vitro* to varying degrees. The induction of TLR5 signaling revealed that flagella were expressed by all the tested strains, but to different extents. Flagella and pili were observed by electron microscopy on the newly isolated strain GRL 1172.

## Introduction

The gastrointestinal tract (GIT) of humans and animals harbors an extremely complex microbiota composing a highly diverse ecological community. The members of the microbiota are either autochthonous (indigenous), being able to permanently colonize the GIT, or transient, possessing abilities for only short-term residence. Many species of the genus *Lactobacillus* are common but subdominant occupants in this community (Guarner and Malagelada, 2003), with varying colonization capacities and, thus, abilities for permanence and crosstalk activity with the host.

*Lactobacillus ruminis*, a member of the *Lactobacillus salivarius* clade (Felis and Dellaglio, 2007), was first isolated from human feces, and has subsequently been commonly detected in other animals, including bovines, pigs, and horses (Lerche and Reuter, 1960; Sharpe et al., 1973; Al Jassim, 2003; Yin and Zheng, 2005; O’Donnell et al., 2015). Particularly in pigs, *L. ruminis* is one of the dominant lactic acid bacteria in the large intestine (Al Jassim, 2003; Yin and Zheng, 2005). Moreover, it was found to be one of the few known intestine-dwelling lactobacilli that represents a true autochthonous member of the GIT microbiota of humans and animals (Tannock et al., 2000; Reuter, 2001).

For the autochthonous members of lactobacilli and of the microbiota in general, colonization of the host GIT is dependent on effective multiplication and/or adhesive capacities. It has been convincingly shown that various cell surface structures of bacteria, such as S-layers and hair-like appendages (pili), are involved in the interactions with the host cells by promoting adherence to the epithelial lining (Hynönen et al., 2002; Kankainen et al., 2009; Turroni et al., 2013; Yu X. et al., 2015). In the genus *Lactobacillus, L. ruminis* is one of the few motile and piliated species (Salvetti et al., 2012). Flagella may facilitate the penetration of the mucus layer by *L. ruminis*, and via its pili and other cell surface molecules, this bacterium may subsequently come into direct contact with intestinal epithelial cells, leading to its colonization and intimate interactions with the host. Furthermore, these colonization properties can also be considered as competitive advantages, and they may partly explain the potential for autochthony of *L. ruminis*. A large number of studies have indeed proven that pili, existing in both Gram-negative and Gram-positive bacteria, are important for mediating host–microbe interactions (Sauer et al., 2000; Rendón et al., 2007; Turroni et al., 2013). In particular, pili are crucial in adhesion to epithelial cells and host extracellular matrix (ECM) proteins, biofilm formation, and the modulation of innate immune responses (Danne and Dramsi, 2012). For instance, the SpaCBA pili of *Lactobacillus rhamnosus* GG (LGG), one of the best characterized lactobacilli with proven probiotic effects, promote strong adhesive interactions with Caco-2 cells and human intestinal mucus, whereas the closely related but non-piliated *Lactobacillus rhamnosus* Lc705 is clearly less adhesive (13). Moreover, LGG with SpaCBA activates the Toll-like receptor 2 (TLR2)-dependent NF-κB signaling pathway to produce proinflammatory and anti-inflammatory cytokines (von Ossowski et al., 2010, 2013; Lebeer et al., 2012; Douillard et al., 2013). Similarly, our recent *in vitro* studies have demonstrated that the LrpCBA pili of *L. ruminis* ATCC 25644 enhance bacterial adaptation to the intestinal niche by mediating strong binding to fibronectin and type I; collagen, Caco-2, and HT-29 cells, and by inducing immunosuppression via the inhibition of NF-κB activation and IL-8 production (Yu X. et al., 2015). In contrast, whole *L. ruminis* cells induce immunostimulating responses via the TLR2 or flagellin-TLR5 signaling pathways *in vitro* (Neville et al., 2012; Yu X. et al., 2015). Additionally, *L. ruminis* cells have been shown to have stimulatory effects on the secretion of tumor necrosis factor (TNF) by using the human monocytic cell line THP-1 (Taweechotipatr et al., 2009). Even though TNF is known to mediate inflammation and immune function, it has also been suggested to possess desirable properties, such as anti-infection and antitumor activities (Dinarello, 2003; Calzascia et al., 2007). Thus, a slight enhancement of proinflammatory cytokine secretion may be advantageous for the host (Neville and O’Toole, 2010). Therefore, *L. ruminis*, with potential immunostimulatory effects, has been considered as a potential immunoprobiotic candidate (Taweechotipatr et al., 2009). Meanwhile, a recent study demonstrated that *L. ruminis* SPM0211 showed antiviral effects by inhibiting rotavirus replication in Caco-2 cells and in a neonatal mouse model (Kang et al., 2015).

The epithelial barrier prevents the entry of pathogens and harmful molecules to host tissues, and epithelial tight junctions (TJ), consisting of a complex of proteins (e.g., occluding and claudins), are the crucial structures functioning as a barrier between adjacent epithelial cells (Furuse et al., 1998). Lactobacilli have the potential to maintain the barrier integrity in the GIT, as accumulating *in vitro* and *in vivo* studies have demonstrated that some indigenous or probiotic *Lactobacillus* strains can prevent the loss of barrier integrity caused by intestinal pathogens (Roselli et al., 2007; Karczewski et al., 2010; Liu et al., 2015; Yu Q. et al., 2015). For instance, LGG efficiently protects the epithelial barrier *in vitro* from disruption induced by enterohemorrhagic *Escherichia coli* O157:H7 by counteracting the redistribution of tight junction proteins (Johnson-Henry et al., 2008). However, the effects of *L. ruminis* on the epithelial barrier have so far been unknown.

To date, *L. ruminis* has remained an insufficiently characterized autochthonous gut bacterium with poorly known functions. Thus far, genomes of only a few *L. ruminis* strains have been sequenced (Forde et al., 2011; Lee et al., 2011; O’Donnell et al., 2015). Based on the few available studies, the fermentation patterns, stress survival abilities, and flagellum-mediated motilities of *L. ruminis* isolates of human, bovine, horse, and pig origin are strain dependent (O’Donnell et al., 2011; Neville et al., 2012; O’Donnell et al., 2015). To expand knowledge of strain characteristics within *L. ruminis* and to facilitate host–microbe interaction studies, new genome-sequenced isolates are needed. In this study, we successfully isolated a novel *L. ruminis* strain, GRL 1172, from porcine feces and observed its surface structures by electron microscopy. We also analyzed the pathogen– and host–microbe interactions of the new isolate and of three other *L. ruminis* strains (ATCC 25644, ATCC 27780, and ATCC 27781), originating from different mammalian hosts, by investigating the adhesion of the strains to ECM proteins and intestinal epithelial cell lines, and by examining their abilities to inhibit the adherence of selected pathogens to ECM proteins or epithelial cells. In addition, we studied the growth inhibition of the selected pathogens by *L. ruminis* culture supernatants and determined whether the *L. ruminis* strains can improve the integrity of the epithelial barrier or protect intestinal epithelial cells against ETEC infection *in vitro*. Finally, we explored the innate immune responses induced by the four *L. ruminis* strains and their culture supernatants in human embryonic kidney (HEK) 293 cells overexpressing TLR2 or TLR5 receptors.

## Materials and Methods

### Sampling, Isolation, and Identification of *L. ruminis* of Porcine Origin

A fecal sample from a 3.5-year-old sow, commercially bred in Southern Finland, was taken during rectal palpation for pregnancy diagnosis. The sow was reared according to standard practices for animal care, and permission to obtain fecal samples was granted by the owner. The sow was healthy and not pregnant at the moment of sampling. Its diet consisted of barley, oats, and a commercial supplementary fiber-rich feed (Tiineys-Pekoni Kuitumix, Suomen Rehu). The fecal sample obtained was immediately placed in an anaerobic jar with an anaerobic atmosphere (AnaeroGen) and brought to the laboratory within 1 h. An aliquot of 5 g was mixed with 45 ml of pre-reduced buffered peptone water (LAB 204) in a Stomacher bag including a filter (BagPage^®^) and homogenized for 1 min with a Stomacher instrument (EasyMIX^TM^ AES Laboratoire). After this, 10 μl spots of the filtrate were plated in the centers of semisolid de Man Rogosa Sharpe (MRS) (Difco) plates with 0.4% (w/v) agar. The plates were incubated anaerobically at +37°C for 66 h. If a turbid zone was observed surrounding the inoculated spot, a small amount of bacterial mass from the edge was picked into 1 ml of MRS broth and incubated anaerobically at +37°C for 24 h. The motile isolates were pure cultured on MRS agar, Gram stained, tested for catalase, and stored at -70°C in MRS broth containing 15% glycerol.

Based on motility, the Gram-positive, rod-shaped, non-sporing cell morphology and the negative catalase test, one isolate, named GRL 1172, was selected for DNA-based identification. The genomic DNA of GRL 1172 was extracted using the Wizard^®^genomic DNA purification kit (Promega). A 4.1-kb region in the LrpCBA pilus operon (Yu X. et al., 2015) was amplified with oligonucleotide primers 5′-CGATTGTTTTTGCAATGGGGAAGTTCGAC-3′ and 5′-GATCGAAGAATTTACCTTGTTTCCGGTCTG-3′ (Oligomer Oy, Finland). The nearly complete 16S rRNA gene was amplified with a universal primer pair (5′-CTGGCTCAGGAYGAACGCTG-3′ and 5′-AAGGAGGTGATCCAGCCGCA-3′) (Koort et al., 2004), purified with the Qiaquick PCR purification kit (Qiagen), and sequenced at the Institute of Biotechnology (University of Helsinki, Finland). The 16S rRNA sequence obtained was compared with the 16S rRNA gene sequence of *L. ruminis* ATCC 27780 (Åvall-Jääskeläinen, unpublished data) and with the NCBI GenBank 16S rRNA sequence database, using the BLAST program with the blastn algorithm on the NCBI Web server^1^. In addition, we have genome sequenced GRL 1172 (Kant et al., 2017).

### Bacteria and Growth Conditions

The bacteria used in this study are listed in **Table 1**. The *L. ruminis* strains were cultivated in MRS broth or on MRS agar plates overnight or for 2 days, respectively, at +37°C under anaerobic conditions. *E. coli, Salmonella enterica*, and *Yersinia enterocolitica* strains were cultured in tryptic soy broth (TSB, Difco) and *Listeria monocytogenes* in brain heart infusion broth (BHI, Difco) with agitation at +37°C overnight.

**Table 1:** List of bacterial strains used in this study.

	Strain	Origin or reference
*Lactobacillus ruminis*	GRL 1172	Pig (this work)
	ATCC 25644	Human
	ATCC 27780	Bovine
	ATCC 27781	Bovine
Pathogens	*Escherichia coli* ETEC (F4^+^)	Pig (Roselli et al., 2007)
	*Escherichia coli* ATCC 43894 (EHEC, O157)	Human
	*Escherichia coli* ERF 2014 (F18^+^, O141)	Pig (Hynönen et al., 2014)
	*Salmonella enterica* serotype *typhimurium* ATCC 14028	Chicken
	*Yersinia enterocolitica* DSM 13030	Human
	*Listeria monocytogenes* ATCC 19117	Sheep

### Cell Lines

HEK293-derived HEK-TLR2 and HEK-TLR5 cells (InvivoGen), harboring human TLR2 and TLR5 genes, respectively, and a secreted embryonic alkaline phosphatase (SEAP) reporter gene placed under the control of an NF-κB and activating protein (AP)-1-inducible promoter, were cultured in Dulbecco’s modified Eagle’s medium (DMEM) (Gibco) supplemented with 10% (v/v) heat-inactivated fetal calf serum (FCS) (Sigma), 50 U/ml penicillin (Sigma), 50 μg/ml streptomycin (Sigma), 2 mM L-glutamine (Sigma), and 100 μg/ml Normocin^TM^ (Sigma). Caco-2 cells were cultivated in RPMI1640 medium (Gibco) supplemented with 10% FCS, 25 mM HEPES buffer, 1% non-essential amino acids, 2 mM L-glutamine, and 50 μg/ml gentamicin (all from Sigma). The HT-29 cell line, purchased from the European Collection of Authenticated Cell Cultures, was maintained in McCoy’s 5A modified media with 10% FCS, 2 mM L-glutamine, 50 U/ml penicillin, and 50 μg/ml streptomycin. IPEC-1 cells were cultured in DMEM/Ham’s F12 (1:1) (Pan Biotech), supplemented with 5% non-heat inactivated FCS, 15 mM HEPES buffer, 1% insulin-transferrin-sodium selenite supplement (BD) and 20 μg/ml epidermal growth factor (BD). All the cells were cultivated at 37°C in 5% CO_2_. When these cells reached approximately 90% confluence, they were seeded and used in the experiments.

### Electron Microscopy

Negative staining and immunolabeling with rabbit anti-pilin antisera against LrpA, LrpB, and LrpC of *L. ruminis* ATCC 25644 were performed as previously described (Chang et al., 2013). The samples were examined under a JEM-1400 transmission electron microscopy (JEOL, Ltd.).

### Adhesion Experiments

#### Bacterial Adhesion to Extracellular Matrix (ECM) Proteins

Human ECM proteins, including fibronectin, type I, and type IV collagen (Sigma), were coated at 100 μl/well on Maxisorp 96-well microtiter plates at the concentration of 10 μg/ml by overnight incubation at +4°C. A volume of 100 μl/well of 2% (w/v) bovine serum albumin (BSA) (Sigma) was added as a negative control. Before adding the bacteria, the wells were blocked with 2% BSA at room temperature for 2 h, followed by washing three times with PBS. Bacterial cells, metabolically labeled with ^3^H-thymidine (Perkin Elmer), were normalized to an OD_600_ of 0.5 (0.5 × 10^9^ cells/ml) in PBS, added at 100 μl/well, and incubated for 2 h at RT. After washing three times with PBS, 100 μl/well of 0.1 mol/L NaOH–1% SDS was added and the plates were incubated at +37°C overnight. The lysed cell suspension was collected into vials containing 1 ml of Optiphase Hisafe III scintillation liquid (Perkin Elmer) and the radioactivity was measured as previously described (von Ossowski et al., 2010). The proportion of adhered cells was determined by comparing the measured radioactivity of the lysed cell suspension with that of the cell suspension added to the wells.

#### Bacterial Adhesion to HT-29 Cells

HT-29 cells were seeded into 24-well plates at a cell density of 10^4^ cells/well and cultivated for 14 days at +37°C in 5% CO_2_. After washing the wells once in Dulbecco’s phosphate buffered saline (DPBS), 600 μl/well of ^3^H-thymidine labeled *L. ruminis* cells, adjusted to an OD_600_ of 0.5, was added and incubated for 1 h at +37°C in 5% CO_2_. The wells were washed three times with DPBS, 600 μl/well of 0.1 mol/L NaOH–1% SDS was added, and the plates were incubated overnight at +37°C in 5% CO_2_. The following steps were the same as those described for ECM binding tests.

#### Inhibition of Pathogen Adherence by *L. ruminis*

The inhibition experiments were conducted by comparing the adherence of pathogens, metabolically labeled with ^3^H-thymidine, to different targets with and without *L. ruminis*. A pathogen/*Lactobacillus* ratio of 1:10 measured by optical densities (OD_600_ = 6.0 for *L. ruminis*, OD_600_ = 0.6 for pathogens) was used in all experiments. Three experimental set-ups were used: competition (simultaneous addition of *L. ruminis* and pathogen cells to the target), exclusion (preincubation of the target with *L. ruminis* with or without the removal of *L. ruminis* and washing before the addition of pathogens), and displacement (preincubation of the target with pathogens followed by the removal of the pathogens and washing before the addition of *L. ruminis*). The different experimental set-ups are detailed below.

The inhibition of F4^+^ ETEC adherence to IPEC-1 cells by *L. ruminis* was tested as previously described (Hynönen et al., 2014), except that the *L. ruminis* strains were cultivated on MRS plates for 2 days, and then collected and directly suspended in DMEM/Ham’s F-12 medium (1:1) (without supplements) to normalize the cell densities. The inhibition of pathogen adherence to Caco-2 cells was tested as previously described for IPEC-1 cells (Hynönen et al., 2014), with slight modifications: the *L. ruminis* strains were suspended to RPMI 1640 medium (without supplements) to normalize the cell densities, the Caco-2 cell layers on 96-well plates were washed only three times at the end of the experiment, and the volume of 1% SDS in 0.1 M NaOH used to lyse the cells was 100 μl. In both exclusion and displacement assays, lactobacilli or pathogen cells, respectively, were removed after 1 h of incubation, and the Caco-2 cell layer was washed once with PBS before the addition of the second bacterial species (pathogen or *L. ruminis* in exclusion or displacement assays, respectively).

To test the inhibition of pathogen adherence to ECM proteins, the wells of 96-well plates were coated with type I collagen or fibronectin by adding 1 μg of protein/well in 100 μl of PBS and by incubating overnight at room temperature, after which the coating solutions were discarded and the wells were washed once with 200 μl of PBS. The wells were then blocked with 2% BSA in PBS (200 μl/well) for 2 h at room temperature and washed three times with PBS (200 μl/well). The bacteria were cultivated as for the inhibition assays with Caco-2 cells and suspended in PBS. The protocol then followed that used in the inhibition studies with Caco-2 cells. Additionally, in each experiment, three wells coated with 2% BSA in PBS without any added bacteria were used to determine the background radioactivity, and three wells per pathogen to determine the non-inhibited adherence of the pathogens to BSA as controls.

### Inhibition of Pathogen Growth by the Culture Supernatants of *L. ruminis*

*Lactobacillus ruminis* strains were cultured for 20 h and the ability of the filter-sterilized (0.2 μm) culture supernatants to inhibit the growth of selected pathogens was tested in a turbidometric assay, as previously described (Skyttä and Mattila-Sandholm, 1991; Hynönen et al., 2014). The pH values of the supernatants were adjusted to the pH of plain MRS (6.2) or left unadjusted (pH 4.1–4.3), and stored in aliquots at -20°C. For each pathogen investigated, three independent experiments with three replicates for each supernatant were performed. As controls, the pathogens were grown without *L. ruminis* culture supernatants. The inhibition caused by the supernatants was computed using the area under the growth curve (AUC) gained during the first 12 h of growth, automatically processed by Research Express software (Transgalactic, Ltd.), and expressed as the area reduction percentage (ARP), as described earlier (Lähteinen et al., 2010). The relationship between the ARP values and colony forming unit (CFU) counts was estimated with linear regression, as previously described (Skyttä and Mattila-Sandholm, 1991).

### Effects of *L. ruminis* on the Barrier Function of Caco-2 Cells

#### Measurement of *trans*-epithelial Electrical Resistance (TEER) and Fluorescein Isothiocyanate-dextran (FITC-dextran) Permeability

Caco-2 cells were seeded at 5 × 10^4^ cells on the membranes of Thincert cell culture inserts with a 1-μm pore size (Greiner Bio-One) and cultivated for 21 days to achieve differentiation. Overnight-cultured bacteria were harvested, washed once in DPBS, resuspended in growth media for Caco-2 with all the other supplements but no antibiotics, and normalized to an OD_600_ of 0.5. After measuring the TEER value using a Millicell ERS-2 Voltohmmeter (Millipore), 140 μl of LAB [multiplicity of infection (MOI) 800] or 28 μl of ETEC (MOI 40) were added per insert and incubated at +37°C in 5% CO_2_. After 24, 48, and 72 h of incubation, the TEER value was recorded. The percentage changes of TEER were calculated by comparing to the baseline (TEER value before adding the bacteria).

When the Caco-2 cells had been incubated with the bacteria as described above for 30 min, 100 μl/insert of FITC-dextran (10 kMW, Thermo Scientific) at the concentration of 300 μg/ml was added to the apical side of the inserts, and the cells were incubated at +37°C in 5% CO_2_. After 4, 8, 21, and 48 h of incubation, 50 μl of basolateral medium was collected into a black 96-well plate (Thermo Scientific) and the fluorescence of the FITC-dextran permeated from the apical to the basolateral side was measured using a Victor3 fluorimeter (Perkin Elmer).

#### Immunofluorescence Assay of Tight Junction Proteins

When Caco-2 cells had been incubated with the bacteria as described above without FITC-dextran for 11 h at +37°C in 5% CO_2_, the cells were washed four times with PBS, and then fixed with 200 μl/insert of -20°C methanol for 5 min, followed by an additional four washes with PBS. One drop/insert of Image-iT FX signal enhancer (Life Technologies) was added and the cells were incubated for 30 min at room temperature to block the unspecific binding sites. After washing with PBS, the cells were incubated with 100 μl/insert of rabbit anti-occludin (1:500; Invitrogen), rabbit anti-ZO-1 (1:100; Thermo Scientific), or mouse anti-claudin-4 (1:1000; Invitrogen) antibodies diluted in PBS at room temperature for 1 h, washed four times with PBS, and then incubated with Alexa Fluor 488 goat anti-rabbit IgG (1:1000; Invitrogen) or Alexa Fluor 594 goat anti-mouse IgG (1:1000; Invitrogen) diluted in PBS at room temperature for 1 h. After washing four times with PBS, the Thincert membranes were cut off and mounted on glass slides in Fluoprep medium (BioMérieux). The cells were observed for tight junction proteins using fluorescence microscopy (Leica DM400B).

### Stimulation of HEK-TLR2 and HEK-TLR5 Cells by *L. ruminis* and its Culture Supernatants

HEK-TLR2 and HEK-TLR5 cells were seeded into 24-well plates (Corning) at the density of 5.0 × 10^4^ cells per well. For HEK-TLR5 cell experiments, the bacteria were grown on MRS plates for 2 days, while for HEK-TLR2 cell experiments the bacteria were cultured overnight in MRS broth. HEK293 cells were stimulated with bacteria with a MOI of 100 bacteria per cell (MOI 100), and the production of SEAP in the culture supernatants was measured on the following day: 180 μl/well of pre-warmed QUANTI-Blue reagent (InvivoGen) was added to 96-well plates (Corning) containing 20 μl of the culture supernatant, followed by 1 h incubation at +37°C. The production of SEAP was measured at 620 nm using a microplate reader (Thermo). IL-8 in the culture supernatants was measured with the BD OptEIA^TM^ ELISA kit (BD Biosciences) according to the manufacturer’s instructions. The experiments with heat-killed bacteria or with Transwell membrane inserts with 0.4-μm pores (BD Biosciences) were performed as previously described (von Ossowski et al., 2013).

In stimulation experiments with bacterial culture supernatants, buffer exchange of the culture supernatants was first accomplished according to the manufacturer’s instructions (Bio-Rad). Briefly, bacteria cultured overnight in MRS broth were centrifuged and the bacterial supernatant was collected. Three milliliters of each supernatant was passed through an Econo-Pac 10DG column pre-equilibrated with the culture medium for HEK293 cells. To elute the column, 4 ml of the same HEK293 medium was loaded and collected. To sterilize the buffer-exchanged bacterial supernatant, the collected eluent was filtered through a 0.45-μm polyethersulfone membrane (VWR). Of the bacterial supernatant obtained, 50 μl was added to HEK-TLR2 cells, and the production of SEAP in the culture supernatants was measured on the following day as described above. The effect of heat was studied by heating the filter-sterilized supernatants at 100°C for 2 h before incubation on HEK293-TLR2 cells.

## Results

### Isolation and Identification of *L. ruminis*

The presumptive motile phenotype of *L. ruminis* (Sharpe et al., 1973; O’Donnell et al., 2015) was exploited in this study to isolate *L. ruminis* from porcine feces. Our isolation protocol yielded several motile isolates (results not shown), of which GRL 1172 was selected for further identification, as its morphological and biochemical characteristics resembled those of *L. ruminis*. As the PCR amplification of a 4.1 kb region in the *lrpCBA* pilus operon yielded a product of the expected size (results not shown), the 16S rRNA gene of GRL 1172 was subsequently sequenced. In the BLAST analysis, the sequence of the 16S rRNA gene of GRL 1172 possessed over 99% similarity with that of *L. ruminis* strains ATCC 27780, NBRC 102161, and ATCC 27782 (99.72, 99.72, and 99.65%, respectively), while the type strain of the phylogenetically closest other *Lactobacillus* species, *L. apodemi* DSM 16634^T^, showed only 93.86% similarity in its 16S rRNA gene sequence, confirming the identification of GRL 1172 as *L. ruminis*.

### GRL 1172 is Flagellated and Piliated

**FIGURE 1:**
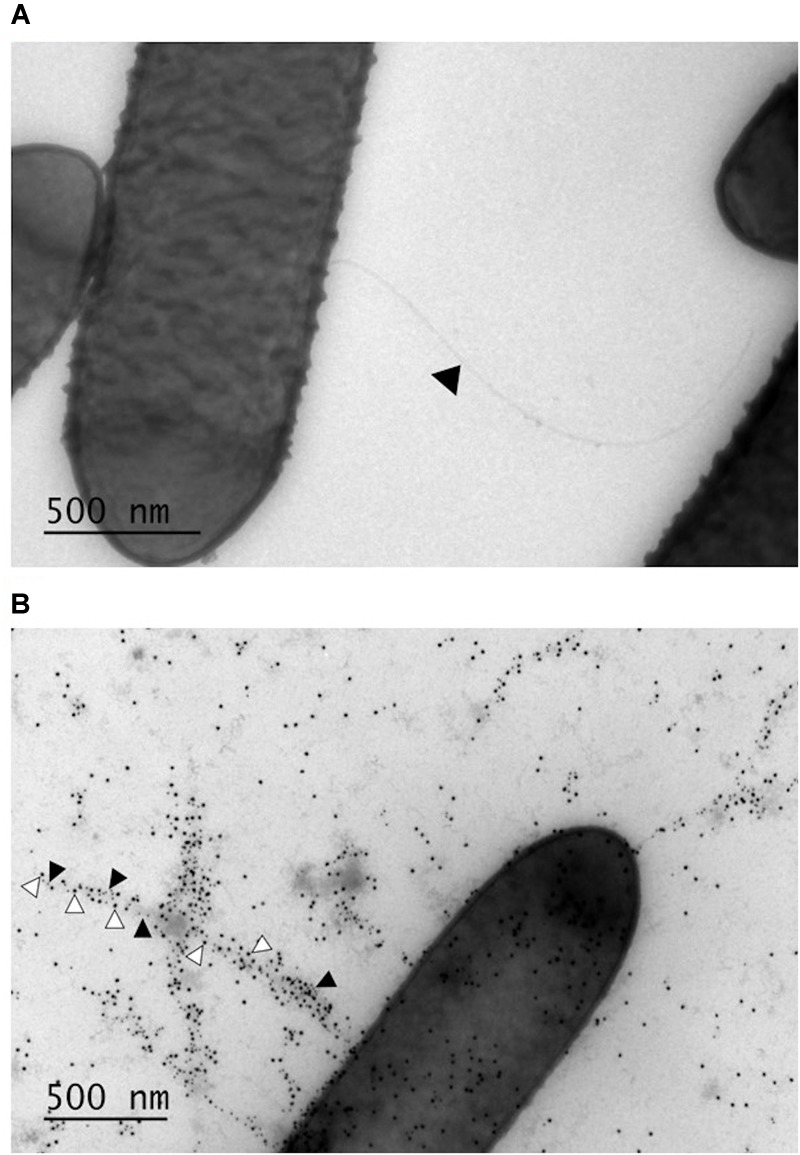
**Surface structures of GRL 1172 visualized by electron microscopy. (A)** Negative staining of GRL 1172. The arrowhead indicates a flagellum. **(B)** Immunoelectron microscopy of GRL 1172. GRL 1172 cells were treated with rabbit anti-pilin antisera against the main pilus subunit LrpA, the tip pilin LrpC, and protein A coupled with gold. LrpA is visualized by small gold particles (black arrowheads) and LrpC by large gold particles (white arrowheads).

Given that GRL 1172 was motile on MRS agar, we assumed that it was flagellated, and its surface structures were subsequently analyzed by electron microscopy. Negative staining by methylcellulose-uranyl acetate solution confirmed that GRL 1172 was indeed flagellated (**Figure 1A**), with one or several flagella protruding from the bacterial body. However, bacteria without flagella were also observed. The majority of bacteria expressed multiple pili, as revealed by immunoelectron microscopy with anti-LrpCBA antibodies (**Figure 1B**).

### Adhesion of *L. ruminis* to ECM Proteins

**FIGURE 2:**
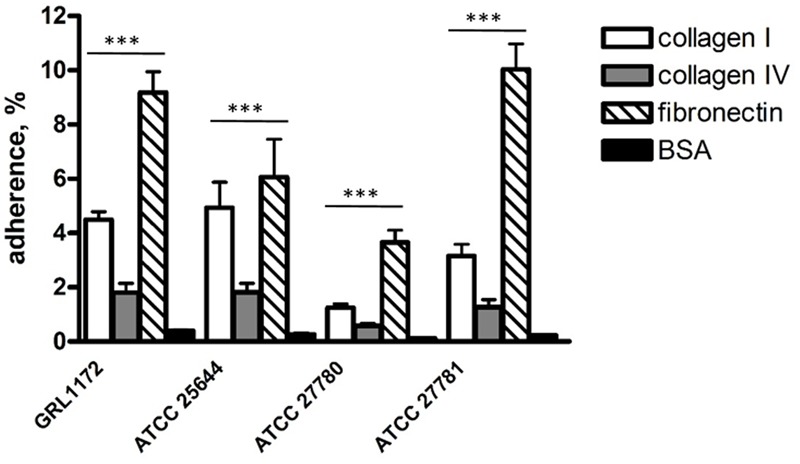
**Adhesion of *Lactobacillus ruminis* strains to ECM proteins.** The bars show the means of five independent experiments, each with three technical replicates, and the error bars express the standard errors of the means (SEM). The statistical differences analyzed by pairwise comparisons against 2% BSA were shown as: ^∗∗∗^*P* ≤ 0.0001 (extremely significant).

Our previous data demonstrated that *L. ruminis* ATCC 25644 of human origin binds to human ECM proteins such as fibronectin, type I, and IV collagen (Yu X. et al., 2015). In this study, we tested whether such binding is a common characteristic in *L. ruminis* isolated from different hosts or whether it is strain-specific. As shown in **Figure 2**, all four *L. ruminis* strains (GRL 1172, ATCC 25644, ATCC 27780, and ATCC 27781) bound to type I collagen and fibronectin. However, the binding efficiencies of the different strains were variable. Our new strain, GRL 1172, bound well to type I collagen and fibronectin, showing rather similar binding levels to these proteins as ATCC 25644 and ATCC 27781, respectively. On the other hand, the bovine strain ATCC 27780 exhibited the weakest adhesive capacities to all the ECM proteins: its type I collagen binding was extremely significantly (*P* ≤ 0.0001) lower than the binding of GRL 1172, ATCC 25644, and ATCC 27781, and its fibronectin binding was extremely significantly (*P* ≤ 0.0001) lower than the binding of GRL 1172 and ATCC 27781, but showed no significant difference (*P* > 0.05) as compared to the binding of ATCC 25644. The adherence of the all *L. ruminis* strains to type IV collagen was low.

### Adhesion of *L. ruminis* to Epithelial Cells

**FIGURE 3:**
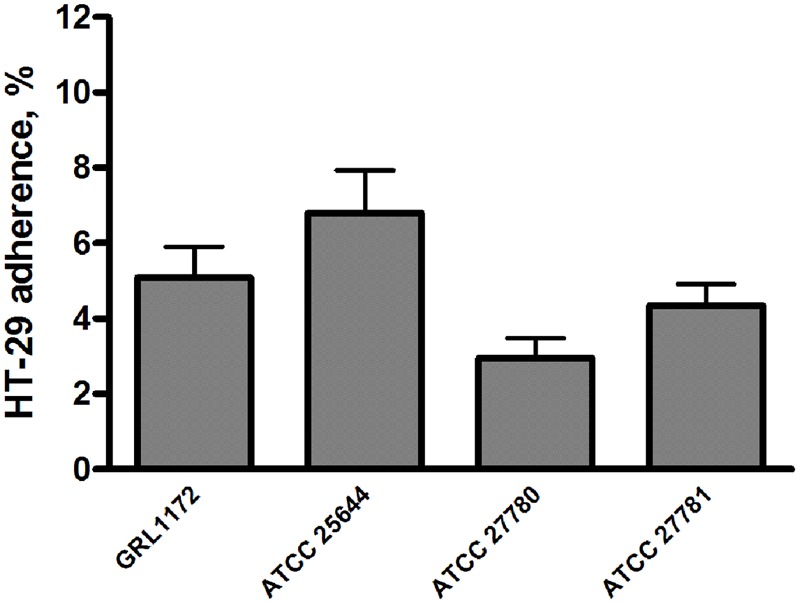
**Adhesion of *L. ruminis* strains to HT-29 cells.** The bars show the means of three independent experiments, each with five technical replicates, and the error bars express the SEM.

We also assessed the adhesiveness of the four *L. ruminis* strains to different intestinal epithelial cell lines. All four strains failed to bind to IPEC-1 cells, and showed poor adherence (0.5–2%) to Caco-2 cells (data not shown). In contrast, all four *L. ruminis* strains tested were able to adhere to HT-29 cells to varying degrees (**Figure 3**). Specifically, ATCC 27780 showed the weakest adherence, while the other three *L. ruminis* strains bound rather strongly to HT-29 cells.

### Inhibition of Pathogen Adherence to ECM Proteins

**FIGURE 4:**
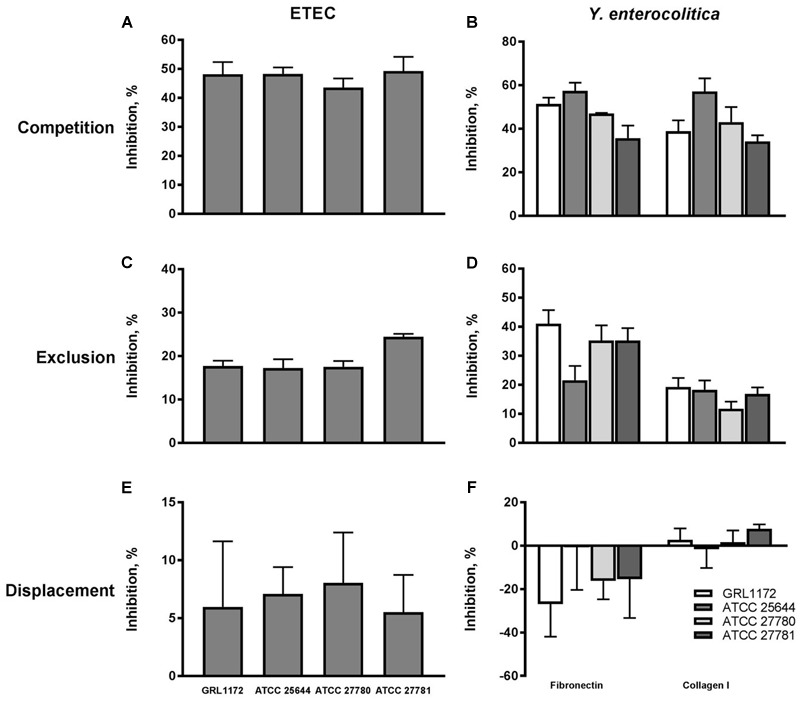
**Inhibition of pathogen adherence to ECM proteins cells by *L. ruminis*.** The inhibition of F4-fimbriated ETEC adherence to type I collagen **(A,C,E)** and the inhibition of *Yersinia enterocolitica* DSM 13030^T^ adherence to fibronectin and type I collagen **(B,D,F)** by *L. ruminis* strains in competition **(A,B)**, exclusion **(C,D)**, and displacement **(E,F)** assays was tested with ^3^H-labeled ETEC and *Yersinia* cells as detailed in Section “Materials and Methods.” The means and standard deviations of three independent experiments are shown, each with three technical replicates.

As the four *L. ruminis* strains adhered efficiently to the human ECM proteins fibronectin and type I collagen (see above), it was of interest to examine whether they could also inhibit the binding of pathogens to the same targets. We chose *Y. enterocolitica* and ETEC as model pathogens and first verified that the *Yersinia* strain used adheres to human fibronectin and type I collagen, and the ETEC strain to type I collagen (results not shown). In competition assays, all the *L. ruminis* strains were able to inhibit the adherence of both pathogens by 35–60% (**Figures 4A,B**). In exclusion assays, the inhibition rates of ETEC and *Y. enterocolitica* were around 20–25 and 15–40%, respectively (**Figure 4C**), and, except for ATCC 25644, all the strains showed a weaker inhibition of *Y. enterocolitica* adherence to type I collagen than to fibronectin (**Figure 4D**). None of the *L. ruminis* strains was able to displace previously adhered pathogens from ECM proteins (**Figures 4E,F**).

### Inhibition of Pathogen Adherence to Epithelial Cells

**FIGURE 5:**
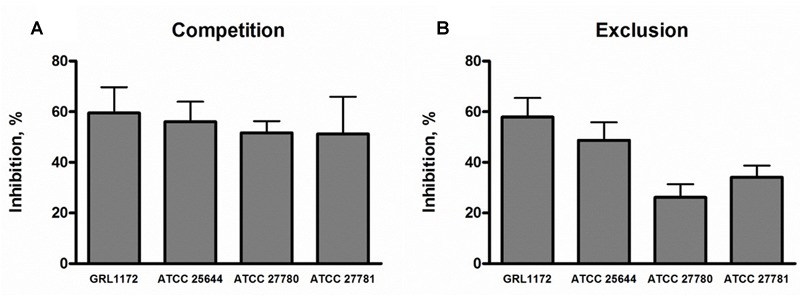
**Inhibition of F4-fimbriated ETEC adherence to IPEC-1 cells by *L. ruminis*.** The inhibition of F4-fimbriated ETEC adherence to IPEC-1 cells by *L. ruminis* strains in competition **(A)** and exclusion **(B)** assays was tested with ^3^H-labeled ETEC cells as detailed in Section “Materials and Methods.” The means and standard deviations of three independent experiments are shown, each with three technical replicates.

The capacities of the four *L. ruminis* strains to inhibit the adherence of ETEC to porcine small intestinal epithelial cells was tested in the IPEC-1 cell model. The ETEC strain used expresses F4 (K88) fimbriae, adheres to IPEC-1 cells (data not shown), and is associated with post-weaning diarrhea in piglets (Jin and Zhao, 2000; Shepard et al., 2012). When added to IPEC-1 cells in 10-fold excess compared to ETEC, all four *L. ruminis* strains were able to reduce the adherence of ETEC by approximately 50–60% in competition assays (**Figure 5A**). If the *L. ruminis* strains were allowed to settle on IPEC-1 cells before the addition of the pathogen (exclusion), a similar level of inhibition (50–60%) by ATCC 26544 and GRL 1172 was observed, while the two bovine strains were slightly less inhibitory (**Figure 5B**).

**FIGURE 6:**
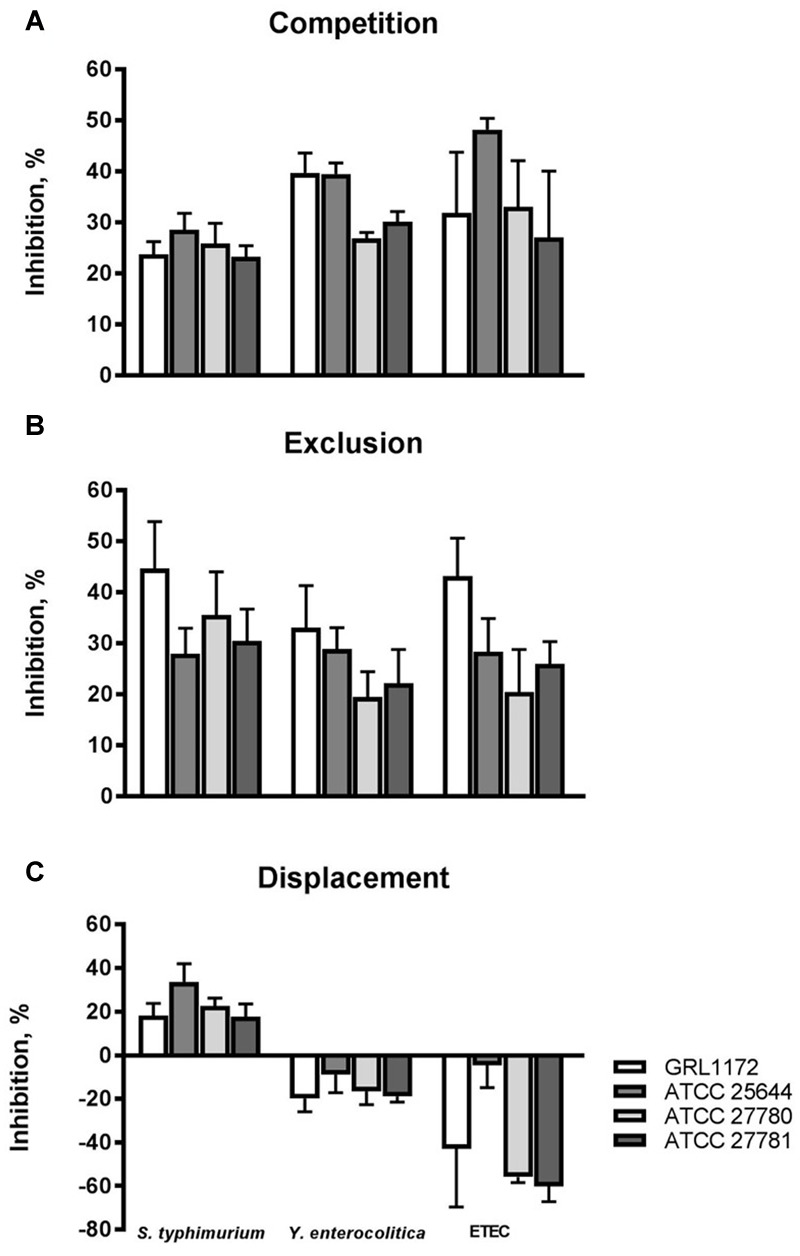
**Inhibition of pathogen adherence to Caco-2 cells by *L. ruminis*.** The inhibition of *Salmonella typhimurium* ATCC 14028, *Y. enterocolitica* DSM 13030^T^ and F4-fimbriated ETEC adherence to Caco-2 cells by *L. ruminis* strains in competition **(A)**, exclusion **(B)**, and displacement **(C)** assays was tested with ^3^H-labeled selected pathogenic cells as detailed in Section “Materials and Methods.” The means and standard deviations of three independent experiments are shown, each with three technical replicates.

We also tested the abilities of the four *L. ruminis* strains to inhibit the adherence of different human or animal pathogens to Caco-2 cells, a commonly used model for human small intestinal epithelium. The capacities of the pathogen strains to adhere to Caco-2 cells was first confirmed (data not shown). All the *L. ruminis* strains were able to inhibit the adherence of *Salmonella typhimurium, Y. enterocolitica*, and ETEC to Caco-2 cells by 20–50% if *L. ruminis* and the pathogens were added simultaneously (**Figure 6A**), and by 20–45% if the *L. ruminis* cells were first pre-incubated on Caco-2 cells and then removed, followed by washing, before the addition of pathogens (**Figure 6B**). Previously adhered *S. typhimurium* cells could even be displaced from Caco-2 cells by all four *L. ruminis* strains, while the displacement of the other pathogens was not observed (**Figure 6C**): the adherence on and/or invasion of Caco-2 cells by *Yersinia* and ETEC was not affected or was even enhanced by the later addition of *L. ruminis*.

### Inhibition of Pathogen Growth by the Culture Supernatants of *L. ruminis*

**FIGURE 7:**
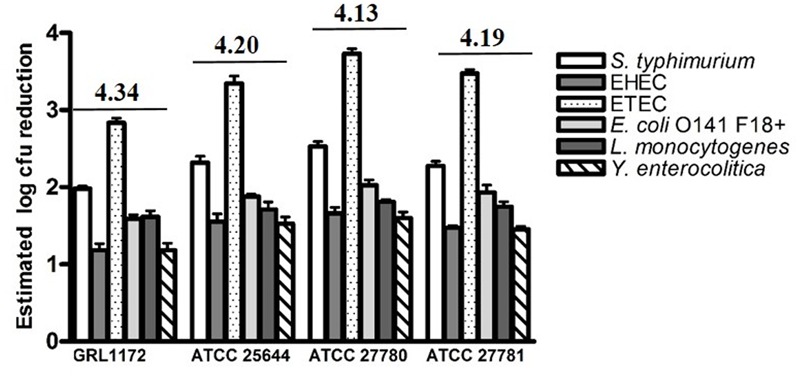
**Reductions in pathogen numbers by *L. ruminis* culture supernatants.** Six different pathogens were cultivated in TSB medium supplemented (10% V/V) with filter-sterilized, non-pH adjusted supernatants of *L. ruminis* strains. The reductions in pathogens numbers, expressed as log colony forming unit (CFU) values, were estimated from the area reduction percentages (ARPs) of the pathogen growth curves by linear regression. The pH value of each supernatant is indicated above the histograms. The means and standard deviations of three independent experiments are shown, each with three technical replicates.

The culture supernatants of the four *L. ruminis* strains were tested for their abilities to inhibit the growth of selected pathogens. All the supernatants inhibited the growth of the pathogens tested (**Figure 7**). The most efficient growth inhibition was noted for ETEC. The supernatants of ATCC 25644, ATCC 27780, and ATCC 27781 reduced the growth of ETEC more than 1000-fold, and a growth reduction of slightly under 1000-fold was noted for the supernatant of strain GRL 1172. The growth of the other pathogens was reduced by approximately 10- to 100-fold. When the pH of the culture supernatant was adjusted to the pH of plain MRS, the growth of the pathogens was lowered by less than 10-fold (data not shown). This suggests that most of the inhibition of pathogen growth in this experiment was due to the acidity of the supernatants, caused by lactic acid produced by the homofermentative *L. ruminis*.

### Effects of *L. ruminis* on the Barrier Function of Caco-2 Cells

The abilities of the four *L. ruminis* strains, GRL 1172, ATCC 25644, ATCC 27780, and ATCC 27781, to maintain the membrane barrier integrity and to prevent membrane damage caused by ETEC were assessed using the human intestinal epithelial cell line Caco-2.

**FIGURE 8:**
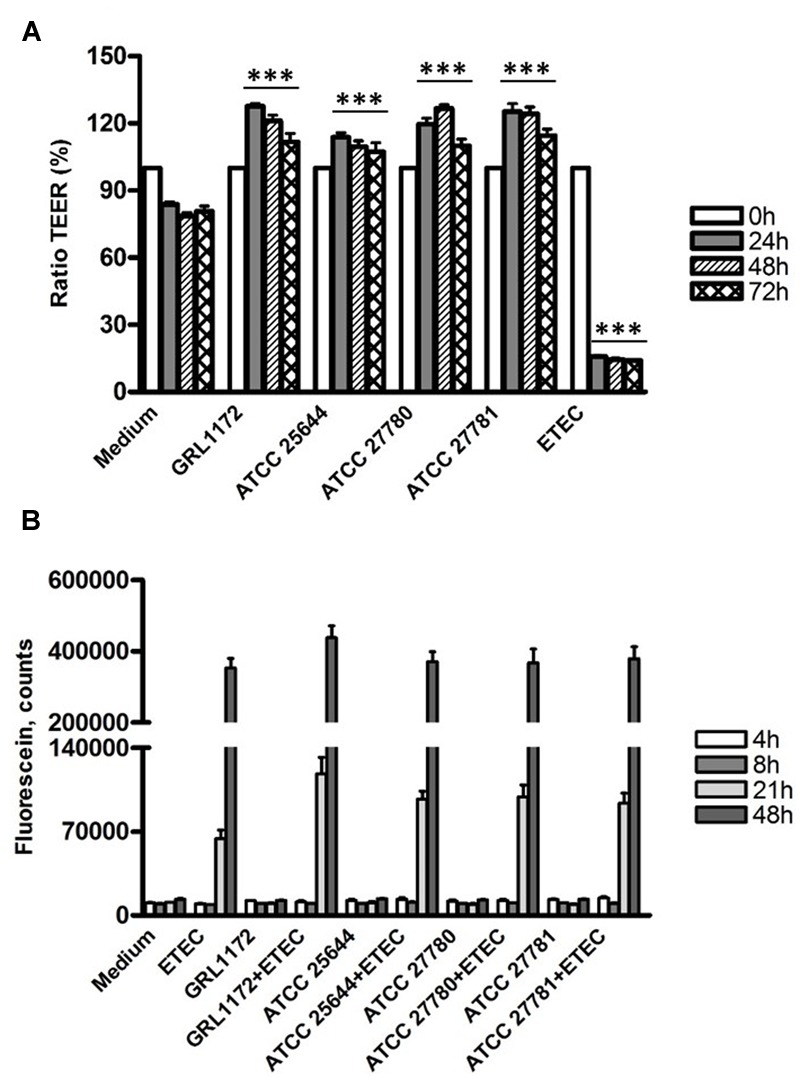
**Changes of barrier function by *L. ruminis*, ETEC and *L. ruminis* with ETEC.** The changes were evaluated by TEER **(A)** and dextran diffusion **(B)**. The bars show the means of three independent experiments, each with two technical replicates, and the error bars express the SEM. The statistical differences analyzed by pairwise comparisons against the negative control (medium) with the same incubation time were shown as: ^∗∗∗^*P* ≤ 0.0001 (extremely significant).

As shown in **Figure 8A**, the TEER values in the Thincerts with Caco-2 cells, incubated with cell culture medium, declined slightly during overnight incubation, which might be due to the presumed slight damaging of the tight junctions during the over 3-week cultivation of Caco-2 cells on Thincerts. However, the values dropped sharply to around 15% when the cells were treated with ETEC. Interestingly, all four *L. ruminis* strains significantly increased TEER values 24 h after the addition of bacteria, followed by small reductions on the third day, but with values still higher than TEER values before adding bacteria. The results indicated that all four *L. ruminis* strains improved the barrier integrity of Caco-2 cells.

This finding was confirmed by the dextran diffusion experiment (**Figure 8B**). When the cells were treated with the cell culture medium only or with *L. ruminis* strains only, there was no dextran diffusion during the whole experimental period. However, abundant diffusion of dextran was detected when ETEC alone or ETEC with *L. ruminis* were incubated on the cells for 21 h, and the diffusion increased drastically at 48 h. These data suggest that even though the *L. ruminis* strains enhanced the cell barrier integrity, they could not protect Caco-2 cells from the damage caused by ETEC.

**FIGURE 9:**
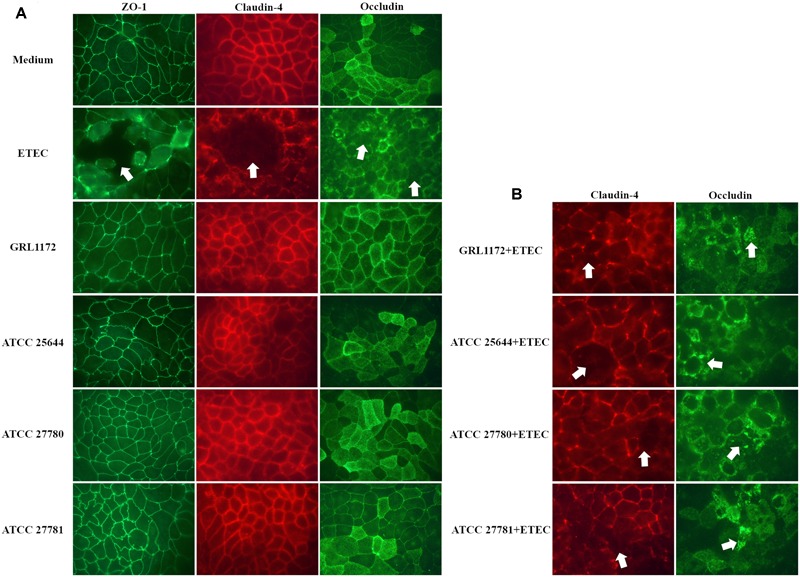
**Immunofluorescence assay of tight junction proteins.** Caco-2 cells were incubated with plain cell culture medium, with ETEC, or with *L. ruminis* strains GRL 1172, ATCC 25644, ATCC 27780, and ATCC 27781 either alone **(A)** or with ETEC **(B)** for 11 h, and an indirect immunofluorescence staining was performed with antibodies specific for the tight junction proteins indicated. The dislocation or loss of the proteins was indicated by arrows. Shown are the results of one representative experiment out of two.

Immunofluorescence assays were performed to study the location of the tight junction proteins during co-culture with ETEC and/or *L. ruminis* (**Figure 9**). When the Caco-2 cells were treated with *L. ruminis* strains alone (**Figure 9A**), the continuous lines between adjacent cells were clearly present, indicating that the locations of ZO-1, claudin-4, and occludin did not change and the epithelial barrier was intact. Conversely, co-incubation of cells with ETEC resulted in the disruption of the cell layer, shown as visible holes between the cells as indicated by arrows in **Figure 9A**. Additionally, the cell boundaries were blurred, and scattered fluorescent dots could be seen inside the cells, indicating that the locations of these proteins had been affected and the barrier had been disordered. Similar results were obtained after incubating ETEC with *L. ruminis* strains on Caco-2 cells (**Figure 9B**). The results again demonstrated that the barrier defects in the tight junctions, caused by ETEC, could not be prevented by the *L. ruminis* strains.

### Pattern Recognition Receptor (PRR)-mediated Immunomodulatory Effects of *L. ruminis*

**FIGURE 10:**
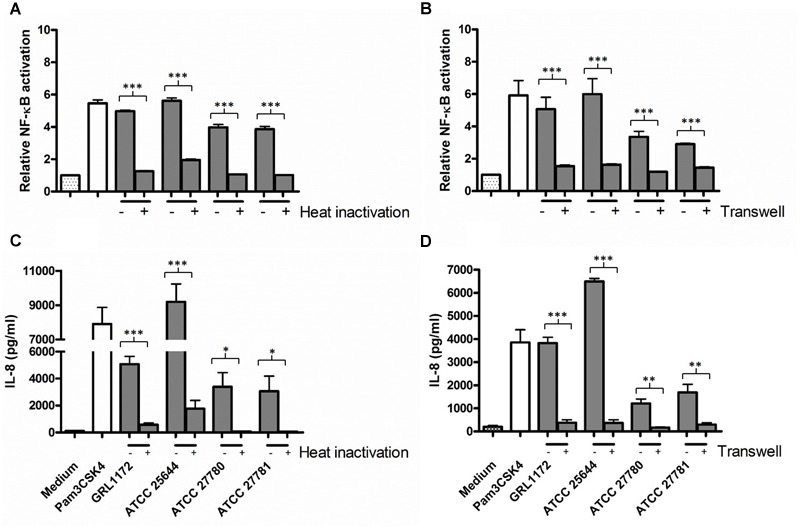
**Induction of TLR2-mediated NF-κB activation and IL-8 production by *L. ruminis* strains. (A,C)** Live (–) or heat-inactivated (+) *L. ruminis* cells were incubated on HEK-TLR2 cells and the secretion of secreted embryonic alkaline phosphatase (SEAP) through NF-κB **(A)** or the production of IL-8 **(C)** was measured as quadruplicates or duplicates, respectively, from the cell culture media according to the manufacturers’ instructions. **(B,D)** *L. ruminis* cells were incubated on HEK-TLR2 cells grown without (–) or grown on (+) Transwell membrane inserts and the secretion of SEAP through NF-κB **(B)** or the production of IL-8 **(D)** was measured as quadruplicates or duplicates, respectively, from the cell culture media according to the manufacturers’ instructions. Pam3CSK4 is a synthetic triacylated lipopeptide and agonist for TLR2. The bars show the means of three independent experiments and the error bars express the SEM. The statistical differences analyzed by pairwise comparisons of samples with or without heat treatment, and samples with or without Transwell were shown as: ^∗∗∗^*P* ≤ 0.0001 (extremely significant), ∗∗*P* ≤ 0.005 (very significant), and ^∗^*P* ≤ 0.05 (significant).

We have previously demonstrated that *L. ruminis* ATCC 25644 induces NF-κB activation and IL-8 induction through TLR2 signaling at a considerably high level (Yu X. et al., 2015). In the present study, we compared the NF-κB activation and IL-8 production of the four *L. ruminis* strains and found that all these strains were able to induce both activities, but to clearly different extents (**Figure 10**). In general, GRL 1172 and ATCC 25644 showed stronger NF-κB activating effects than the bovine ATCC 27780 and ATCC 27781 strains. The levels of NF-κB activation exerted by these two strains were as high as that of the positive control [HEK-TLR2 cells treated with the TLR2-agonist lipopeptide Pam3CSK4 (1 ng/ml)]. To test the putative proteinaceous nature of the immunomodulating component, we simultaneously performed experiments using heat-inactivated bacteria (**Figures 10A,C**). Except for ATCC 25644, heat inactivation almost completely abolished the capacity of *L. ruminis* strains to stimulate NF-κB through TLR2 and the subsequent IL-8 secretion. To further determine whether TLR2-dependent immunomodulatory responses were triggered by direct contact between bacteria and HEK-TLR2 cells, Transwell cell culture membrane inserts with a 0.4-μm pore size were used to separate the cells from the bacteria (**Figures 10B,D**). Without cell-to-cell contact, NF-κB activation and IL-8 production were at the background level.

**FIGURE 11:**
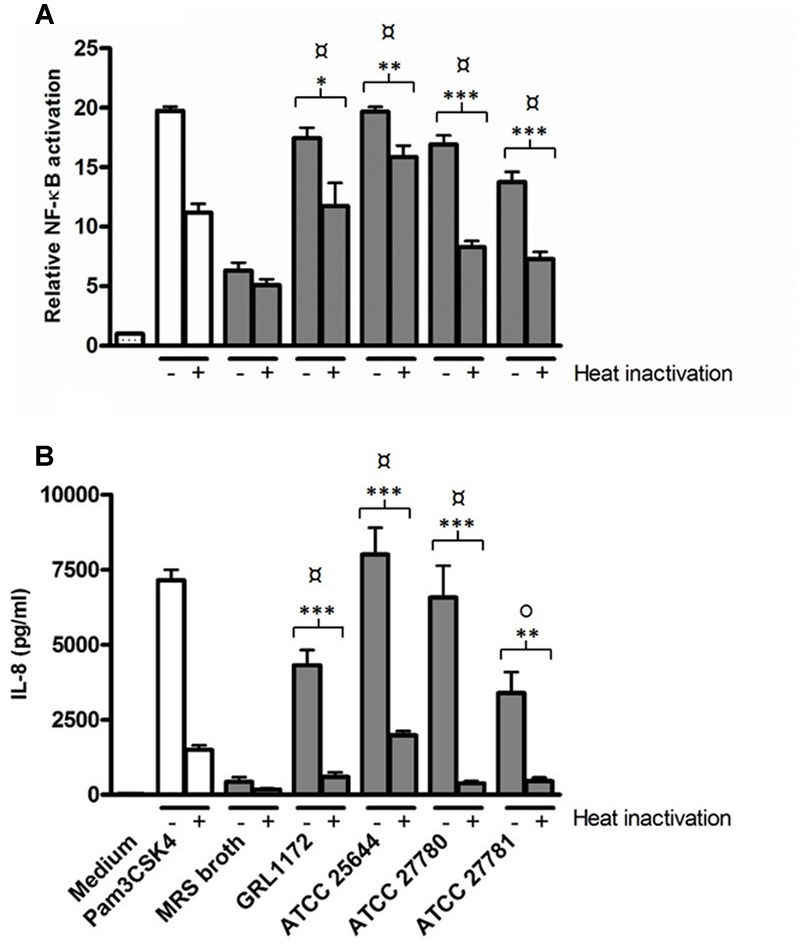
**Induction of TLR2-mediated NF-κB activation and IL-8 production by *L. ruminis* culture supernatants.** Bacterial culture supernatants without (–) or with heat-inactivation (+) were incubated on HEK-TLR2 cells. The secretion of SEAP through NF-κB **(A)** and the production of IL-8 **(B)** were measured as quadruplicates and duplicates, respectively, from the cell culture media according to the manufacturers’ instructions. The bars show the means of three independent experiments and the error bars express the SEM. The statistical differences analyzed by pairwise comparisons against MRS broth were shown as: *P* ≤ 0.0001 (extremely significant), °*P* ≤ 0.005 (very significant). Pairwise comparisons between samples with or without heat treatment were shown as: ^∗∗∗^*P* ≤ 0.0001 (extremely significant), ∗∗*P* ≤ 0.005 (very significant), and ^∗^*P* ≤ 0.05 (significant).

Additionally, we evaluated whether the culture supernatants of the four *L. ruminis* strains have the capacity to activate TLR2 signaling. As **Figures 11A,B** clearly show, all the different bacterial supernatants contained TLR2-stimulating factors. High temperature treatment of the supernatants only slightly lowered NF-κB activation (**Figure 11A**), whereas IL-8 production was drastically reduced (**Figure 11B**).

**FIGURE 12:**
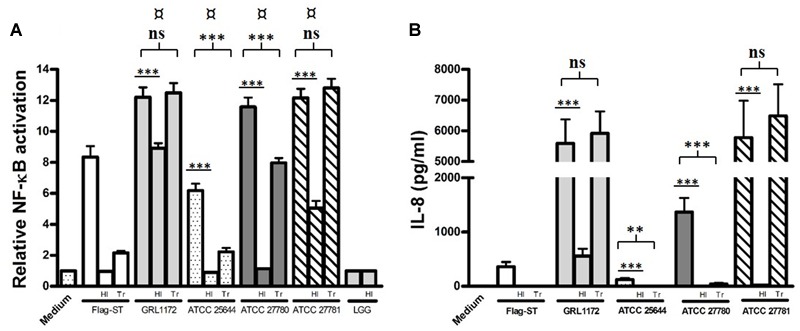
**Induction of TLR5-mediated NF-κB activation and IL-8 production by *L. ruminis* strains.** Live or heat-inactivated (HI) *L. ruminis* cells were incubated on HEK-TLR5 cells, and the SEAP through NF-κB **(A)** and the production of IL-8 **(B)** were measured as quadruplicates and duplicates, respectively, from the cell culture media according to the manufacturer’s instructions. HI, *L. ruminis* was heat-inactivated before incubation; Tr, HEK-TLR5 cells were grown on Transwell membrane inserts; Flag-ST, flagellin isolated from *S. typhimurium*. The bars show the means of three independent experiments and the error bars express the SEM. The statistical differences analyzed by pairwise comparisons against *L. rhamnosus* GG (LGG, an aflagellated strain) were shown as: ¤*P* ≤ 0.0001 (extremely significant). Pairwise comparisons between samples with or without heat treatment, and samples with or without Transwell inserts were shown as: ∗∗∗*P* ≤ 0.0001 (extremely significant), ∗∗*P* ≤ 0.005 (very significant), and ns *P* ≥ 0.05 (not significant).

As immunoelectron microscopy demonstrated that GRL 1172 is flagellated, we further confirmed the finding by using HEK-TLR5 cells, which were treated with *L. ruminis* grown on MRS agar plates for 2 days to induce motility. Indeed, all four *L. ruminis* strains greatly induced TLR5-dependent NF-κB activation, indicating that they are all flagellated (**Figure 12A**). Notably, even though ATCC 25644 elicited NF-κB activation through TLR5 signaling at a level of only 50% less than the other strains (**Figure 12A**), IL-8 secretion induced by ATCC 25644 was considerably lower (**Figure 12B**). Similarly to the TLR2 experiments described above, we performed heat inactivation and Transwell experiments with *L. ruminis* and HEK-TLR5 cells. The abilities of all four *L. ruminis* strains to activate TLR5 were markedly reduced when the cells were inactivated by heat (**Figure 12A**). Except for GRL 1172, none of the heat-inactivated *L. ruminis* strains could trigger IL-8 secretion (**Figure 12B**). In the Transwell system, the *L. ruminis* strains showed active TLR5-mediated NF-κB signaling (**Figure 12A**). GRL 1172 and ATCC 27781 with or without Transwell induced the highest levels of IL-8, while the induction of IL-8 by ATCC 27780 was drastically affected by the Transwell system, although the level still remained significantly higher than that of the negative control (**Figure 12B**).

## Discussion

*Lactobacillus ruminis* is commonly found in humans and many animals as an autochthonous member in the GIT (Tannock et al., 2000; Reuter, 2001). However, the factors contributing to its colonization, autochthony, and interplay with the host are still poorly understood. A recent study indicated that *L. ruminis* has the potential to survive in bile salts and under oxygen-rich conditions, but the survival rate was strain specific (O’Donnell et al., 2015). To broaden knowledge of the characteristics of this species, comparisons between four *L. ruminis* strains with different origins (pig, human, and bovine) were performed in the present study.

Epithelial cells and their underlying ECM form an essential barrier to prevent the entry of external agents or organisms such as autochthonous and pathogenic bacteria. According to some reports, autochthonous bacteria, in healthy individuals, appear to live and multiply in the lumen suspension, separated from epithelial cells by mucus and having no direct contact with them (van der Waaij et al., 2005). However, to explain persistent colonization of the GIT, the adherence of autochthonous bacteria to host cells, mucus, or other proteins is often evaluated (Vélez et al., 2007), and has been considered as one of the niche adaptation factors. The epithelial cells and ECM (exposed in the damaged host mucosa) act as targets for the attachment and colonization of both pathogens and non-pathogenic microorganisms (Štyriak et al., 2003; Rendón et al., 2007; Chagnot et al., 2012). Bacterial surface proteins, such as S-layer proteins (Greene and Klaenhammer, 1994; Antikainen et al., 2002; Hynönen et al., 2002) and sortase-dependent proteins (van Pijkeren et al., 2006; Tripathi et al., 2013; von Ossowski et al., 2013), have been demonstrated to mediate adherence to these targets. For instance, in some pathogens, pili are involved in the interaction with epithelial cells and ECM, further creating a path leading to disease and illness (Telford et al., 2006). On the other hand, the epithelial cell- and ECM-binding abilities mediated by S-layers and pili of indigenous (Lorca et al., 2002) or probiotic microbes (Lebeer et al., 2012; Hynönen et al., 2014) can competitively inhibit pathogen binding, thus preventing or hindering their colonization. Therefore, the investigation of adherence is also of importance when characterizing autochthonous microbes.

In this study, we isolated a new *L. ruminis* strain from the porcine GIT and investigated its adherence to ECM-proteins and to porcine (IPEC-1) and human (Caco-2, HT-29) intestinal epithelial cell lines, in parallel with *L. ruminis* strains of human or bovine origin. All the strains, including the porcine and human ones, bound poorly to the piglet small intestinal cell line IPEC-1 and to Caco-2 cells representing adult human small intestine. As *L. ruminis* is found all over the porcine (Al Jassim, 2003; Yin and Zheng, 2005) and human (Tannock et al., 2000; Reuter, 2001) GITs *in vivo*, these cell lines apparently do not correctly model its *in vivo* adhesion targets in the small intestine. Alternatively, *L. ruminis* may rely on adherence to ECM-proteins or other targets (and/or on efficient multiplication) while colonizing the small intestine, rather than (or in addition to) binding to epithelial cells. In contrast, the efficient adherence of the *L. ruminis* strains, especially the human one, to the colonic HT-29 cells in this study may reflect *L. ruminis* abundance in the human colon (Tannock et al., 2000; Reuter, 2001).

The binding of our *L. ruminis* strains to mucins in the small or large intestine is unlikely, since, based on our previous results, the *L. ruminis* species is an ineffective mucus binder (Yu X. et al., 2015, unpublished results), and its genome does not encode for proteins that share homology with established mucus-binding domains (Kant et al., 2017). Rather instead, *L. ruminis* is more likely to inhabit the epithelial cell zone of the gut, as it has the capacity to bind ECM proteins, primarily by the adhesiveness of its surface piliation, *lrpCBA*- encoded pili in case of strain ATCC 25644 (Yu X. et al., 2015), and we presume that the binding behavior of the other three *L. ruminis* strains is also pilus-dependent. The *lrpCBA* pilus operon has been verified by PCR in ATCC 27780 and ATCC 27781 and, based on genomic data, at least one of the genes encodes a pilin protein with a predicted collagen-binding domain (our unpublished data). Another bovine strain, ATCC 27782, has also been predicted to have a pilus locus and a fibronectin binding protein (Forde et al., 2011). Future studies are needed to characterize the role in adherence of pili of the bovine and porcine strains.

Regarding the strain- and host specificity of adherence in *L. ruminis*, the strain ATCC 27780 in our study showed the weakest adherence to ECM-proteins and to HT-29 cells, while the other bovine isolate, ATCC 27781, exhibited much better binding abilities, demonstrating that the binding pattern is not necessarily related to the strain origin but is strain-specific. The human isolate ATCC 25644 adhered to human intestinal epithelial cells slightly better than *L. ruminis* strains from other origins, but, on the other hand, also the other strains did bind to human HT-29 cells. Our results thus do not support the presence of strict host specificity of adherence in *L. ruminis*. The scarcity of available cell lines representing intestinal epithelia of other animals precludes further conclusions about the host specificity of adherence in *L. ruminis*. Finally, it cannot be ruled out that adhesive phenomena not studied in this work, like different mucus-binding specificities of the strains, though not plausible (see above), might reveal some host-specificity not recognized here.

The binding of an intestinal pathogen to the host small intestinal epithelium is a prerequisite for the initiation the infection. In this study, we first tested the binding of pathogens to the epithelial cell lines used and then chose to the inhibition experiments those pathogen–epithelial cell pairs in which evident binding was seen (results not shown). For instance, in line with the fact that ETEC strains expressing F4, F5, F6, or F18 fimbriae are most often implicated in post-weaning diarrhea of piglets (Jin and Zhao, 2000; Shepard et al., 2012), only the F4-fimbriated ETEC strain bound to the piglet-derived IPEC-1 cells in significant numbers, and the binding of this pathogen only was chosen as the target for inhibition in our IPEC-1 cell experiments. Previous studies have shown that lactobacilli often inhibit pathogen binding *in vitro* (Coconnier et al., 1993; Abedi et al., 2013; Hynönen et al., 2014), and inhibition may even be seen although *Lactobacillus* strains were poorly adhesive (Tuomola et al., 1999; Gueimonde et al., 2006), indicating that competitive binding is not the only mechanism through which the inhibition occurs. With this in mind we tested the abilities of all our *L. ruminis* strains to inhibit the adherence of the chosen pathogens to IPEC-1 and Caco-2 cells, despite the poor IPEC-1 and Caco-2 adherence of our *L. ruminis* strains, and indeed found that all the *L. ruminis* strains inhibited pathogen adherence to these cell lines in competition assays. Similarly, ATCC 27780 was found to inhibit pathogen adherence to fibronectin and type I collagen although the adherence of ATCC 27780 to these proteins was low. The inhibition of pathogen adherence did not therefore correlate with the binding capabilities of the *Lactobacillus* strains and may rather be related to interactions between *L. ruminis* and pathogen cells in the liquid phase. As the *L. ruminis* strains adhered very poorly to IPEC-1 cells, we did not wash them away in exclusion assays before we added ETEC cells, making this experimental set-up partially of the competition type, but with reduced possibilities for ETEC and *L. ruminis* to interact in suspension compared to pure competition experiments. In this type of IPEC-1 exclusion experiment, the bovine strains ATCC 27780 and ATCC 27781 inhibited the adherence of ETEC slightly less efficiently than in pure competition assays (**Figure 5B**), supporting the view that the inhibition of ETEC binding to IPEC-1 cells is not due to competitive adherence, but rather to phenomena occurring in the liquid phase, such as coaggregation, as has been observed with *Lactobacillus casei* ssp. *rhamnosus* GR-1 (Reid et al., 1988). In our preliminary experiments, however, we were unable to demonstrate marked coaggregation between ETEC and *L. ruminis* (data not shown).

Previous investigations have indicated that in displacement assays, inhibition is generally considerably lower than in competition or exclusion assays, suggesting different underlying mechanisms in inhibition (Lee et al., 2003; Gueimonde et al., 2006; Zárate and Nader-Macias, 2006). In general, the ability of lactobacilli to displace pathogens has rarely been observed (Lebeer et al., 2008). In accordance with the above findings, our results here indicated that neither *Y. enterocolitica* nor ETEC could be displaced from epithelial cells or ECM proteins by the tested strains. Unexpectedly, however, the adherence and/or invasion of Caco-2 cells by *Salmonella* was 20–30% less efficient if any of the *L. ruminis* strains had been added to Caco-2 cells after the co-incubation with *Salmonella* as compared to the assay with no *L. ruminis*. Similarly, appreciable but varying degrees of displacement of *E. coli* and *Salmonella* from Caco-2 cells could be observed by LGG and *L. casei* Shirota (Lee et al., 2003). According to Lee et al. (2003) and our results, the extent of displacement, when present, seems to be affected by both *Lactobacillus* and the pathogen strains, and the incubation time of lactobacilli may have an effect. However, the mechanism of this displacement remains to be determined.

One of the mechanisms of pathogen growth inhibition by lactobacilli is the secretion of soluble inhibitory factors, such as antimicrobial agents. There is considerable evidence that many *Lactobacillus* strains from different origins, including humans (Annuk et al., 2003; Kõll-Klais et al., 2005) and animals (Otero and Nader-Macías, 2006; Lähteinen et al., 2010; Santini et al., 2010), exhibit abilities to inhibit pathogen growth, which is in agreement with our present study. Notably, in this study, the inhibition rate gradually dropped with the increasing pH value of the culture supernatants of *L. ruminis*, and the growth of the pathogens was lowered by less than 10-fold when the pH of the culture supernatants was adjusted to that of the plain MRS broth (data not shown), suggesting that the inhibition largely relied on the production of lactic acid. The growth inhibition of pathogens to approximately similar levels by the tested *L. ruminis* strains might at least partly explain the inhibition of pathogen adhesion in IPEC-1 cell assays: the multiplication of ETEC was inhibited in the acidic environment. Genes related to bacteriocin production have been identified in the genomes of ATCC 25644 and ATCC 27782 (Forde et al., 2011), and in this study, the growth medium of ATCC 25644 after 20 h of cultivation may thus contain bacteriocins, contributing to the growth inhibition of pathogens. However, the presence of bacteriocin genes in the other isolates, as well as the expression of the genes under the conditions used, remains to be confirmed.

Caco-2 cells, when completely differentiated, constitute confluent and polarized epithelial layers, and are widely used for modeling the small intestinal barrier (Alsenz et al., 1998). Some *Lactobacillus* strains, including *L. plantarum* (Qin et al., 2009), *L. amylovorus* (Roselli et al., 2016), *L. acidophilus* (Kainulainen et al., 2015), and *L. salivarius* (Putaala et al., 2008), have been shown to enhance or to maintain TJ integrity by elevating TEER values and by preventing the epithelial permeability increase caused by pathogens through enhancing the expression of TJ proteins. Here, in agreement with these studies, we demonstrated that all four *L. ruminis* strains maintained the barrier integrity of Caco-2 cells. On the other hand, they could not counteract the barrier disruption caused by ETEC, as dextran diffusion was not inhibited and TJ proteins were dislocated when *L. ruminis* was co-cultured with ETEC. Even though our results revealed that all four *L. ruminis* strains inhibited the binding of ETEC to Caco-2 cells, a possible explanation for the failure to counteract the barrier damage might be that toxins produced by ETEC disrupted the epithelial barrier integrity during the long co-incubation (11 h) with *L. ruminis*.

Pattern recognition receptors (PRRs) on epithelial or immune cells recognize the microbe-associated molecular patterns (MAMPs) and lead to the activation of innate immune responses. TLRs are the most extensively studied PRRs (Iwasaki and Medzhitov, 2004; Lebeer et al., 2010). Previously, we have demonstrated that *L. ruminis* ATCC 25644 induces profound NF-κB activation and IL-8 production through TLR2 signaling (Yu X. et al., 2015). This study confirmed the results with ATCC 25644 and suggested that this is a common characteristic among *L. ruminis*, as all four strains tested evoked such immune responses at variable levels. The variation in the levels of observed activation might be related to the variable presence and/or structural variation of *L. ruminis* cell surface components, such as lipoteichoic acid (LTA), lipoprotein, and lipopeptides, as these structures are known to contribute to the activation of the innate immune responses in lactobacilli (Wells, 2011). Heat treatment and the Transwell system destroyed the TLR2-activating abilities of *L. ruminis*, indicating that the TLR2-activating microbial components are heat sensitive and anchored to the bacterial surface, possibly being cell surface proteins. Nevertheless, the supernatants of the *L. ruminis* strains were interestingly able to stimulate NF-κB activation and IL-8 production, which could be related to the release of LTA or lipoproteins into the culture supernatants, as demonstrated by previous studies (Jones et al., 2005; Henneke et al., 2008). In line with this, the heat treatment of *L. ruminis* supernatants did not completely abolish NF-κB activation. Thus, the results suggest that, in addition to heat-sensitive cell surface components, some other factors in *L. ruminis*, such as secreted heat-resistant molecules, may induce TLR2 responses.

Based on current knowledge, flagellins are the only factors activating TLR5-mediated gene expression (Gewirtz et al., 2001a; Hayashi et al., 2001). As confirmed by TLR5 activation, all four *L. ruminis* strains in this study are flagellated. This contrasted with a previous study, reporting that ATCC 25644 was aflagellated (Neville et al., 2012). An explanation for the discrepancy could be related to the varying expression of flagella under different conditions (Aldridge and Hughes, 2002). Interestingly, the TLR5-mediated NF-κB induction ability of ATCC 25644 and ATCC 27780 was completely abolished by heat treatment, whereas that of GRL 1172 and ATCC 27781 was not affected. This suggests that the flagellins of GRL 1172 and ATCC 27781 might be more heat resistant. However, the mechanism has not been elucidated. The finding that the Transwell system did not prevent NF-κB induction suggests that either flagellum fragments or soluble flagellins passed through the insert membrane (0.4 μm in pore size), as flagellins can even pass through 0.2 μm pore-size filters (Gewirtz et al., 2001b). In particular, flagella of GRL 1172 and ATCC 27781 might be more easily detached, or these bacterial cells may be more easily broken, leading to the release of more flagellins and consequently resulting in high NF-κB induction and IL-8 production. Our results confirmed that flagella are important for NF-κB activation and IL-8 secretion, but cell-to-cell contact is not necessary, as detached or fragmented flagella could also activate the TLR5 signaling pathway. Notably, GRL 1172, ACTT 27780, and ATCC 27781 induced NF-κB at similar levels, but IL-8 production varied. It has been demonstrated that the TLR5-mediated IL-8 secretion induced by flagellin may also occur through other routes, such as p38 mitogen-activated protein kinase (MAPK) signaling, which is independent of NF-κB activation and controls IL-8 expression post-transcriptionally (Yu et al., 2003; Gewirtz et al., 2004). The three *L. ruminis* strains in our study may thus differ in their abilities to activate the p38 route, leading to varied IL-8 production. Varying flagellin molecular structures of the strains may explain the different inducing activities through either route, as according to previous reports, differences in structural domains of flagellin result in different IL-8 induction (Verma et al., 2005; Im et al., 2009). Finally, the presence of secreted IL-8 binding proteins preventing IL-8 detection in the culture supernatants cannot be ruled out, either.

Taken together, this study dissected and compared the functional characteristics of four *L. ruminis* strains with different origins. The flagellated *L. ruminis* strains tested in the current study possessed some common properties, such as adherence to intestinal epithelial cells and ECM proteins, inhibition of pathogens, and the maintenance of epithelial barrier functions and modulation of immune responses, which most likely contribute to the niche adaptation of *L. ruminis* in the host. However, the extent of these effects differed among *L. ruminis* strains. More specifically, the bovine strain ATCC 27780 exhibited weaker adhesive abilities to ECM proteins than the other strains; however, its ability to inhibit pathogen adherence to this target was similar in comparison with the other strains. Furthermore, ATCC 27780 induced only moderate levels of IL-8 secretion through TLR2, while ATCC 25644, of human origin, was the strain that most efficiently induced IL-8 in HEK-TLR2 cells. Finally, the intracellular pathways of TLR5-mediated IL-8 induction, triggered by flagella, may differ between strains. The findings warrant further studies exploring the mechanisms of intestinal persistence and autochthony of *L. ruminis*. Additionally, as mentioned above, *L. ruminis* isolated from human feces has been shown to inhibit rotavirus replication in the GIT of neonatal mouse (Kang et al., 2015), demonstrating that at least some *L. ruminis* strains indeed have probiotic properties. Moreover, given that *L. ruminis* is an autochthonous member in the GIT and is flagellated and piliated, this species could be employed as an effective carrier for oral DNA vaccines. However, more *in vivo* studies are needed to evaluate the probiotic or vaccine carrier potential of *L. ruminis* in different hosts.

## Author Contributions

AP, UH, IvO, XY, SÅ-J, JK, AL, and JR designed this study. XY, SÅ-J, and UH performed the experiments. XY, SÅ-J, UH, and AP analyzed the data and wrote the paper.

## Conflict of Interest Statement

The authors declare that the research was conducted in the absence of any commercial or financial relationships that could be construed as a potential conflict of interest.

## Footnotes

1 http://blast.ncbi.nlm.nih.gov/Blast.cgi
